# Editorial for the Special Issue on Thin Film Microelectronic Devices and Circuits

**DOI:** 10.3390/mi16020167

**Published:** 2025-01-30

**Authors:** Chengyuan Dong

**Affiliations:** Department of Electronic Engineering, Shanghai Jiao Tong University, Shanghai 200240, China; cydong@sjtu.edu.cn

Thin film microelectronic devices and circuits (TFMDCs), including thin film transistors (TFTs), thin film solar cells (TFSCs), thin film sensors (TFSs), thin film memories (TFMs), etc., are playing more and more important roles in electronic industries, such as integrated circuits, semiconductor displays, medical devices, energy devices, optical sensors, and so on [1,2,3]. One of the outstanding feathers of TFMMDCs is flexibility, which results in some brand-new products and applications, such as flexible displays and memories [4,5,6], wearable cells and sensors [7,8], and stretchable medical devices and systems [9,10]. On the other hand, some TFMDCs with special merits can penetrate into the conventional industries and push them forward. For example, amorphous oxide semiconductor (AOS) TFTs show extraordinarily low leakage currents, and can be combined with the back-end of line (BEOL) of complementary metal-oxide-semiconductor (CMOS) integrated circuits, which benefit their applications in low-power dynamic random access memories (DRAMs) [4]. To realize these interesting applications, TFMDCs are still crying for advancements in fabrication technologies, device physics, circuit designs, and system integrations.

This Special Issue comprises 10 original papers about recent advances in the research and development of TFMDCs. Specifically, three microelectronic devices are covered here: thin film transistors (three papers), two-terminal thin film components (five papers), and optical devices (two papers). These typical studies exhibit the recent advances in this interesting field, which are briefly summarized as follows.

For AOS-TFTs, amorphous InGaZnO (a-IGZO) films are the most popular channel layers, which have already been put into mass production [11]. Therefore, many attempts have been taken to improve their electrical performance and stable properties. In this Special Issue, H. Huang et al. [12] investigate the influence of hydrogen buffer layer on the electrical performances of top-gate IGZO-TFTs, indicating that the hydrogen content of the buffer layer increases proportionally with the rise in the hydrogen content of the reaction gases during plasma-enhanced chemical vapor deposition (PECVD); in addition, with the increase in the hydrogen-containing materials in the reactive gases, the field-effect mobility and negative bias illumination stress (NBIS) stability of the corresponding IGZO-TFTs improve nearly twofold.

Gate insulators also exhibit strong effects on the electrical properties of IGZO-TFTs [13]; high-k metal oxides are gradually replacing the traditional SiO_2_ dielectric layer in the new generation of microelectronic devices. In this Special Issue, J. Liu et al. [14] report the production of five-element high entropy metal oxide (HEMO) dielectric films by the solution method, analyzing the role of each metal oxide in the system by characterizing the film’s properties. The IGZO-TFTs with (AlGaTiYZr)O_x_ dielectric layers show a mobility of 182 cm^2^/Vs, a threshold voltage of −0.2 V, and a subthreshold swing of 0.3 V/dec, which implies a good prospect for applying HEMOs to TFT devices.

In addition to a-IGZO, the other AOS channel layers for thin film transistors have also been being studied [15]. In this Special Issue, Z. Wu et al. [16] performed an annealing study on the praseodymium-doped indium zinc oxide (PrIZO) TFTs, which indicates that the channel films tend to be denser and obtain a lower surface roughness, a narrower optical-band gap, and less oxygen-vacancy defects for the higher annealing temperatures; additionally, the PrIZO-TFTs annealed at 150 °C exhibit a desired performance, as well as good flexible properties.

The solution-processed dielectric films can be used as not only the gate insulators of TFT devices, but also the core layers of metal–insulator–metal (MIM) and metal–insulator–semiconductor (MIS) capacitors [17,18,19]. In this Special Issue, X. Fang et al. [20] utilized a low-temperature self-exothermic reaction based on the solution method to prepare high-performance Al_2_O_3_ dielectric thin films on the flexible substrates; the corresponding MIM devices demonstrate excellent electrical performances, including a leakage current density as low as 1.08 × 10^−8^ A/cm^2^ @1 MV and a relative dielectric constant as high as 8.61 ± 0.06.

There is another report relating MIM capacitors in this Special Issue. T. M. Choi et al. [21] investigated the MIM capacitors with different thicknesses of SixNy film and varying levels of film quality to improve their capacitance density. In this study, the C-V characteristics are divided into two categories: the voltage coefficient of capacitance (VCC) and the temperature coefficient of capacitance (TCC). When the thickness of the SixNy film decreases, the VCC increases, whereas the TCC does not vary significantly; the most influential factor for capacitance uniformity is the thickness uniformity of the SixNy film.

The novel dielectric films for MIS capacitors are also reported in this Special Issue. J. U. Yoo et al. [22] proposed a new method to effectively fabricate a poly(vinylidene fluoride) (PVDF)-based TiO_2_ dielectric layer via electrospinning. Improved electrical properties are observed with increasing TiO_2_ anatase content, and the residual amount of PVDF decreased with increasing annealing temperature; the corresponding MIS capacitors annealed at 600 °C exhibit a lower leakage current density of 7.5 × 10^−13^ A/cm^2^ when Vg = 0 V.

Memristor is one of the most interesting two-terminal thin film devices, which might be put into wide applications in the future [23]. However, more systematic studies on the applications of memristors are still necessary at the present stage. In this Special Issue, Y. Kim et al. [24] employed a study of weight quantization associations over a weight range for application in memristors. To minimize the information corruption in the system caused by weight quantization, the concept of “weight range” was introduced, which has a direct impact on weight, reducing the number of digits represented by a weight below a certain level.

MoS_2_ films have been attracting much attention due to their interesting properties [25]. There is an interesting study about MoS_2_ and its device by pulsed laser deposition in this Special Issue [26]. The authors gradually increased the pulsed laser energy density from 70 mJ·cm^−2^, and finally prove that 100 mJ·cm^−2^ is the best-pulsed laser energy density. The Si/MoS_2_ heterojunction prepared under the optimized laser energy density indicates an opening voltage of 0.61 V and reaction ratio of 457.0.

Lately, thin film optical devices are playing more and more important roles in the actual applications [27,28]. In this Special Issue, two reports regarding interferometers and light-detection-sensors are presented. J. Liao et al. [29] propose a polarization-insensitive lithium niobate-on-insulator (LN) interferometer, and make further improvements. By elaborately designing the geometric structure of the multimode interference coupler, beam splitting of equal proportions and directional coupling of higher-order modes are realized. At 1550 nm, the visibility of the interferometer is 97.59% and 98.16% for transverse electric (TE) and transverse magnetic (TM) modes, respectively, demonstrating the high performance of the proposed LN polarization-independent interferometer.

Traditional light-direction angle sensors relying on optical components like gratings and lenses face inherent constraints form diffraction limits in achieving device miniaturization. In this Special Issue, P. Huang et al. [30] proposed a sensor concept capable of discerning the 3D light-direction based on a segmented concentric nanoring structure, which is sensitive to both elevation angle and azimuth angle at a micrometer device scale. The concept was validated through simulation studies. This design broadens the angle sensing dimension based on mutual resonance coupling among nanoring segments, and through waveguide implementation or sensor array arrangements. The detection range can be flexibly adjusted to accommodate diverse application scenarios.

Finally, I would like to thank all of the authors for submitting their papers to the Special Issue “Thin Film Microelectronic Devices and Circuits”, as well as all the reviewers and editors for their contributions to improving these submissions.
